# Benign Envy in Nursing: A Qualitative Exploration of Perceived Triggers and Drivers

**DOI:** 10.1155/jonm/2916301

**Published:** 2026-09-29

**Authors:** Mehdi Golverdi, Mohammad Sadegh Sharifirad

**Affiliations:** ^1^ Faculty of Management and Accounting, College of Farabi, University of Tehran, Tehran, Iran, ut.ac.ir; ^2^ Department of Humanities and Social Sciences, Faculty of Public Administration, Ardakan University, Ardakan, Iran, ardakan.ac.ir

**Keywords:** benign envy, drivers, nursing, qualitative research, relatable excellence, social comparison, triggers

## Abstract

**Background:**

Benign envy, a constructive form of upward social comparison, has received limited attention in nursing despite its potential relevance to professional learning, motivation, and growth. Although quantitative research has highlighted the potentially constructive aspects of benign envy, qualitative understanding of how nurses experience, interpret, and respond to benign envy within professional contexts remains limited.

**Purpose:**

This qualitative exploratory study sought to explore the perceptions benign envy within clinical and professional contexts. The research specifically focused on the perceived triggers and drivers described in participants’ accounts and how nurses interpreted and responded to these experiences within clinical and professional contexts.

**Methods:**

Semistructured, in‐depth interviews were conducted with 25 purposively selected nurses from three hospitals in Golestan Province, Iran. Data were analyzed using reflexive thematic analysis.

**Results:**

The analysis developed eight themes concerning participants’ perceived triggers of benign envy and three themes concerning perceived drivers of their responses to the experience. The eight themes for triggers were outstanding professional competencies; financial benefits and income; career advancement and position; leadership and personality qualities; positive moral and behavioral virtues; existential‐spiritual awareness; proactive striving for advancement; and effective therapeutic communication skills. These themes reflected the diverse professional, interpersonal, moral, material, and existential qualities that participants associated with experiences of benign envy. Three further themes concerned drivers of participants’ constructive responses were self‐evaluation coupled with intrinsic motivation; professional modeling coupled with constructive competition; and ethically and spiritually rooted influences.

**Conclusions:**

Benign envy was described as a multifaceted experience embedded in nurses’ professional, relational, moral, and existential contexts. It is sustained by strong ethical frameworks and self‐regulatory processes, enabling nurses to transform upward comparisons into motivation rather than distress. The findings provide a contextual account of how nurses make sense of benign envy and how this experience may be associated with professional growth and constructive responses.

**Implications for Nursing Management:**

The findings suggest several areas through which nurse managers may support constructive responses to upward comparison in nursing workplaces: Transparent career pathways, structured mentorship programs, and recognition of professional, interpersonal, and moral excellence may provide contexts in which nurses can learn from colleagues they admire. Managers may also consider incorporating opportunities for reflective discussion and peer learning to help nurses interpret professional comparison as an opportunity for development rather than interpersonal threat. Attention to nurses’ ethical and professional values may further support the environments in which constructive competition and professional modeling can occur without raising conflicts in relationships. These implications are derived from participants’ accounts and should be considered as practice considerations for further evaluation rather than as tested interventions.

## 1. Introduction

In the high‐stakes nursing profession, hierarchical structures and team dynamics frequently create opportunities for upward social comparisons, rendering envy a prevalent emotion that influences career perceptions and patient care [1]. Envy arises when an individual perceives a personal deficiency relative to another’s superior quality, achievement, or possession and desires to attain that attribute or wishes for the other to lose it [2]. Nurses commonly experience envy toward colleagues due to disparities in resources, promotions, or accomplishments. Malicious envy can disrupt communication and organizational behavior [3, 4], whereas benign envy offers substantial motivational potential [5–8]. Recent cross‐sectional studies indicate that malicious envy intensifies perceptions of career stagnation—particularly hierarchical plateaus linked to institution type and professional choice—while benign envy fosters a constructive drive to enhance skills and performance in order to reach comparable success [1].

A scoping review highlights the relational character of envy in nursing, tracing its origins to social comparisons and its outcomes from interpersonal tension to motivational drives, yet notes limited attention to harnessing benign envy’s benefits [7]. Employees experiencing benign envy display positive emotional responses and genuine respect for peers’ accomplishments [3] while reporting increased self‐efficacy, pointing to pathways for positive adaptation [5]. This duality warrants deeper examination of envy’s constructive dimension in nursing contexts. Quantitative studies have shown that benign envy is positively associated with self‐efficacy [5], task performance [9], and job engagement [10] and that perceived attainability and organizational justice have been associated with benign envy [11, 12]. However, these studies neither capture the subjective meanings nurses attach to their experiences of benign envy nor explain how benign envy unfolds in the specific social and emotional contexts of clinical practice—gaps that a qualitative approach is well positioned to address.

Benign envy serves as a motivational force that encourages skill refinement, performance improvement, and professional advancement through emulation of admired achievements, while generating positive emotions and authentic admiration for peers’ successes [3]. It promotes prosocial behaviors such as offering help, seeking knowledge, and providing communal support [13] and intensifies effort to acquire others’ advantages or accomplishments [14]. Drawing on nurses’ accounts of workplace benign envy, this study addressed the following research questions, framed inductively and consistent with reflexive thematic analysis: (1) How do nurses describe and interpret benign envy in their professional practice? (2) What situational contexts and characteristics do nurses associate with benign envy? (3) What internal cognitive‐motivational influences do nurses describe in relation to constructive responses to benign envy? Triggers represent situations or characteristics participants associated with benign envy [15], while drivers constitute individual appraisals that sustain benign envy as constructive action [16, 17]. Throughout this study, we use “triggers” to refer to observable workplace events or characteristics that elicit the initial envious response and “drivers” to refer to the internal cognitive‐motivational influences that participants described in relation to their constructive responses.

This study makes three contributions. First, although research on benign envy remains limited and predominantly quantitative (e.g., [15, 18–20]), qualitative explorations are scarce. Quantitative approaches, while valuable, often lack depth in complex social dynamics. Qualitative methods provide a strong basis for uncovering underexplored aspects of organizational behavior. This study addresses the gap by applying qualitative techniques to investigate perceived triggers and drivers in healthcare, particularly nursing. Second, prior work has insufficiently examined individual‐level factors related to employees’ accounts of benign envy. This research illuminates motivational drivers and illustrates how individual differences shape workplace emotional responses. Third, the study offers a contextually grounded thematic account of benign envy triggers and their implications within the nursing community.

## 2. Theoretical Background

Benign envy, in contrast to malicious envy that provokes hostility and sabotage, constitutes an adaptive response to upward social comparisons that stimulates genuine self‐improvement motivation without resentment [16, 21]. This distinction has been established through mixed‐methods studies based on narrative recall of envy episodes although foundational qualitative work (e.g., [15]) has been largely overshadowed by quantitative validations that reduce subjective experience to scales and correlations.

Previous research suggests that benign envy is more likely to occur when individuals compare themselves with others who have desirable and attainable advantages in personally important areas, such as career advancement, professional skills, or relationships, particularly when these advantages are perceived as deserved, fair, and attainable [1, 5, 11, 12, 22]. When the envied advantage appears reachable and justly earned, the response shifts from threat to inspiration, often accompanied by admiration, gratitude, and prosocial impulses. Prior research has identified several factors associated with benign envy, including perceived control, organizational justice, a secure self‐concept, emotional self‐efficacy, and cultural norms that emphasize collective growth [23, 24]. In organizational and nursing contexts, resource disparities, career plateaus, salary gaps, and visible colleague successes have been closely associated with its occurrence, especially under supportive leadership and resilient team dynamics [1, 5, 11, 12, 25]. Most studies, however, remain cross‐sectional or rely on retrospective narratives, leaving the dynamic temporal process of benign envy largely unexplored.

Qualitative themes in consumer behavior, social media, and Generation Z (Gen Z) samples similarly identify curated success displays as salient antecedents, moderated by aspirational mindsets, digital literacy, and collectivist values [5, 24]. The field’s reliance on Western and Chinese samples raises questions of cultural generalizability, particularly where religious or collectivistic values (e.g., in Muslim societies) appear to favor benign interpretations [1, 26].

The adaptive potential of benign envy is well established; nevertheless, the qualitative evidence base remains fragmented and concentrated in particular cultural contexts, with relatively limited research using inductive approaches to explore how benign envy is understood in nursing. This study addresses these limitations by providing an in‐depth, contextually grounded exploration of perceived triggers and individual drivers of benign envy among Iranian nurses, where high‐stakes environments and cultural norms may uniquely shape the emotion.

## 3. Method

### 3.1. Research Design

Reflexive thematic analysis, following Braun and Clarke [27–29], was used within a qualitative exploratory design to identify and interpret patterns of meaning related to the perceived triggers and drivers of benign envy in participants’ accounts. These triggers and drivers were not prespecified constructs but were developed inductively as interpretive themes from the interview data.

### 3.2. Participants

Nurses were selected as the focus due to the first author’s clinical experience and professional relationships in Golestan Province hospitals, which revealed that nurses frequently respond to benign envy situations in constructive rather than destructive ways. This observation motivated exploration of their workplace experiences in the province. Twenty‐five nurses from various roles across three hospitals in Golestan Province, Iran, participated. Purposive sampling [30] selected participants who (1) had experienced workplace benign envy, (2) possessed at least 2 years of departmental experience, and (3) were willing to share their experiences openly.

To operationalize this criterion, we first obtained contact lists from hospital administrators to establish initial access to potential participants. During this first interaction, the interviewer provided a standardized, clear verbal explanation of benign envy—distinguishing it from malicious envy by emphasizing that it involves admiration for a colleague’s success and motivates self‐improvement rather than hostility or resentment. Following this explanation, nurses were explicitly asked whether they had ever experienced such positive, constructive feelings toward a colleague. Only those who confirmed having such experiences were then invited to participate in the formal in‐depth interview and provided oral informed consent prior to each session.

Participants ranged from 25 to 45 years of age (19 men and 6 women) and held roles including matron, nurse supervisor, head nurse, intensive care unit (ICU) nurse, staff nurse, rotation nurse, and obstetrical nurse. While Table 1 reports age in broader categories to protect participants’ privacy and adhere to cultural norms regarding personal information, all individuals strictly met the 25–45 age criterions. Table 1 presents detailed demographic characteristics.

**TABLE 1:** Participant demographic characteristics.

Interview	Gender	Age	Job position	Interview duration (minutes)
1	Male	20–30 years	Staff Nurse	43
2	Male	30–40 years	Nurse Supervisor	32
3	Male	40–50 years	Head Nurse	34
4	Male	20–30 years	Staff Nurse	34
5	Male	30–40 years	Rotation Nurse	41
6	Female	20–30 years	Obstetrical Nurse	52
7	Male	30–40 years	Nurse Supervisor	37
8	Male	20–30 years	ICU nurse	29
9	Male	20–30 years	Nurse Supervisor	43
10	Male	20–30 years	Rotation Nurse	44
11	Female	40–50 years	Matron	36
12	Male	20–30 years	Staff Nurse	40
13	Male	30–40 years	Staff Nurse	33
14	Female	30–40 years	Obstetrical Nurse	47
15	Male	30–40 years	Staff Nurse	46
16	Male	20–30 years	Staff Nurse	32
17	Male	40–50 years	Staff Nurse	24
18	Male	20–30 years	Staff Nurse	37
19	Female	20–30 years	Staff Nurse	48
20	Male	30–40 years	Staff Nurse	34
21	Female	20–30 years	Staff Nurse	41
22	Male	30–40 years	Staff Nurse	33
23	Male	20–30 years	Staff Nurse	36
24	Male	30–40 years	Staff Nurse	43
25	Female	30–40 years	Staff Nurse	39

### 3.3. Data Collection

Data collection occurred over 2 months. Initial access to potential participants was facilitated through contact lists provided by hospital administrators, after which participants were selected based on the relevance and richness of their experiences, as established during preinterview conversations. The first two interviews involved nurses with whom the first author had prior professional familiarity. Participants were encouraged to seek clarification and confirm understanding of questions. Face‐to‐face interviews lasted 24–52 min. A semistructured protocol with literature‐derived primary questions was used to guide the interviews, supplemented by follow‐up probes. Table 2 lists all interview questions. Oral informed consent was obtained before audio‐recording. The first author reviewed recordings and transcripts iteratively. Twenty‐five interviews were conducted, providing a rich and varied dataset for reflexive thematic analysis.

**TABLE 2:** Semi‐structured interview protocol.

Question type	Question number	Question objective	Interview question
Primary	P1	To begin the conversation and invite participants to describe situations in which they experienced envy toward a colleague.	Have you ever experienced envy toward a colleague? If so, could you describe the situation, including what happened and how you responded, in detail, please?
Primary	P2	To explore how participants distinguished benign envy from other forms of envy and who or what was the focus of their envy.	Toward which colleagues have you felt benign (positive) envy, and what was the most common subject of this envy? Why?
Primary	P3	To explore the characteristics, achievements, behaviors, or situations that participants linked to feelings of benign envy.	What specific characteristics, achievements, behaviors, or situations of these colleagues prompted feelings of benign envy?
Primary	P4	To explore how participants understood benign envy and what they felt contributed to experiencing it rather than malicious envy.	Why did you tend to feel benign envy rather than malicious (negative) envy toward them?
Primary	P5	To explore how participants described the effects and implications of benign envy in their professional and personal lives.	What were the consequences of experiencing benign envy for you, and how did it impact your professional life, work environment, and personal life? Could you elaborate, please?
Primary	P6	To explore how participants responded to benign envy and what they were hoping to achieve through those responses.	What actions did you take when you felt benign envy toward someone? What were you attempting to achieve?
Follow‐up	F1	To explore participants’ responses to benign envy in greater detail.	Can you walk me through the specific steps you took after recognizing those feelings of positive envy?

### 3.4. Data Analysis

Both authors conducted and transcribed the interviews verbatim. Data were analyzed using reflexive thematic analysis following Braun and Clarke’s approach [29]. The first author maintained a reflexive journal throughout data collection and analysis, reflecting on preconceptions and their potential influence on the analytic process. The analysis involved familiarization with the transcripts, generation of initial codes, development and review of candidate themes, refinement and naming of themes, and development of an interpretive thematic account. Coding was conducted inductively, allowing patterns of meaning to be developed from participants’ accounts rather than being imposed by prior theory. The first author initially developed the codes and candidate themes, and the second author contributed to analytic discussions and refinement of the thematic structure. The analysis yielded 36 codes and 11 themes (Table 3).

**TABLE 3:** Data structure of triggers and drivers of benign envy among nurses.

Thematic domain	Themes	Number of supporting excerpts	Selected codes
Perceived triggers of benign envy among nurses	Outstanding professional competencies	6	Colleague’s discipline and precision
		5	Colleague’s educational background
		8	Colleague’s professional expertise and proficiency
		5	Supervisor’s mastery and competence
		6	Professor’s expertise and skill
		3	Colleague’s crisis management skills
	Financial benefits and income	7	Colleague’s salary
		5	Supervisor’s compensation
		7	Colleague’s occupational income
	Career advancement and position	5	Colleague’s career promotion
		6	Colleague’s job position
	Leadership and personality qualities	6	Supervisor’s composed and dignified personality
		2	Supervisor’s personality maturity
		3	Colleague’s personality maturity
		5	Supervisor’s charisma
	Positive moral and behavioral virtues	7	Colleague’s moral virtue
		3	Colleague’s patience and resilience in workplace adversity
		1	Colleague’s helpfulness
	Existential‐spiritual awareness	3	Colleague’s death awareness
		5	Colleague’s worldly and spiritual attentiveness
	Proactive striving for advancement	7	Colleague’s proactivity and diligence
		4	Colleague’s goal‐oriented efforts
		3	Colleague’s drive for learning and self‐improvement
	Effective therapeutic communication skills	5	Colleague’s patient communication skills
		4	Colleague’s popularity among patients

Perceived drivers of benign envy in nurses	Self‐evaluation coupled with intrinsic motivation dynamics	7	Self‐efficacy belief
		6	Feelings of personal inadequacy
		4	Perceived need for change
		2	Acceptance of interpersonal differences
	Professional modeling coupled with constructive competition	8	Success modeling (emulating others’ success)
		6	Professional behavior modeling
		3	Positive trait emulation
		5	Healthy competitive drive
		3	Positive attitude toward comparison target
	Ethical and spiritual influences	6	Ethical family teachings
		4	Religious/spiritual beliefs

### 3.5. Trustworthiness

Trustworthiness was established through four strategies aligned with Lincoln and Guba’s [31] criteria. Credibility was supported through peer review (three assistant professors in organizational behavior and social psychology reviewed the analysis). The first author maintained a reflexive journal throughout the analytic process to document reflections and consider the influence of preconceptions on the analysis. Dependability was addressed through an audit trail documenting all analytical decisions and transcripts. The authors engaged in analytic discussions regarding codes and theme development, contributing to refinement of the thematic structure. Transferability was supported by thick description of the participants’ characteristics, the hospital contexts, and rich verbatim quotes, enabling readers to assess the applicability of findings to other settings. Maximum variation sampling was achieved by including participants from three different hospitals and diverse professional positions, which further supported transferability. All analytical decisions were documented in an audit trail to support dependability. Additionally, the study design and reporting were guided by the key principles of the Consolidated Criteria for Reporting Qualitative Research (COREQ) checklist [32], particularly regarding research team reflexivity, study design, data collection, and analysis.

### 3.6. Ethical Considerations

This research was approved by the Ethics Committee of Ardakan University with the ethics code IR.ARDAKAN.REC.1405.008. The study was conducted in accordance with the Declaration of Helsinki. Before the study began, all participants were thoroughly informed about the study’s objectives. The nurses were assured that their participation was voluntary and would not affect their employment. Oral informed consent was obtained from all participants prior to each interview session, and all participants were assured of their right to withdraw at any time without consequence.

## 4. Findings

After the analysis, 11 themes concerning benign envy among nurses were developed: eight themes related to situations and qualities that participants associated with experiences of benign envy, and three themes related to perceived drivers of their constructive responses.

### 4.1. Perceived Triggers of Benign Envy Among Nurses

#### 4.1.1. Outstanding Professional Competencies

Outstanding professional competencies emerged as a prominent theme associated with experiences of benign envy. Nurses observed peers or superiors displaying exceptional clinical abilities reflecting rigorous training, attention to detail, and advanced problem‐solving. This theme specifically encompasses clinical and technical skills directly related to patient care—such as diagnostic accuracy, procedural expertise, and crisis management—rather than interpersonal or communicative abilities. Many reported inspiration from colleagues exhibiting superior judgment or refined technical skills. Participant 9 described: *“Yes, I had this feeling of benign envy toward one of my colleagues*… *She was very intelligent and always made accurate diagnoses. I truly admired how well she performed, even though I couldn’t at first. Initially, I tried to distance myself, but eventually, I approached her and expressed my wish to improve. She kindly offered to help by introducing me to a gynecologist’s clinic where I could gain more experience. We ended up working together, and now we are close colleagues and friends.”*

#### 4.1.2. Financial Benefits and Income

Participants associated financial benefits and income with experiences of benign envy. Rather than resentment, this awareness motivated pursuit of legitimate income‐enhancement pathways. One participant explained: *“I admired both her work ethic and her income level. She works hard to maximize her earnings, which aligns with my own goals. When I asked if she could introduce me to the private nursing service she works with, she gladly agreed. Apparently, they earn significantly more by charging per nursing service, which can substantially boost one’s income.”*

#### 4.1.3. Career Advancement and Position

Career advancement and position were associated with benign envy through participants’ observations of higher status and associated respect. These disparities inspired reflection on personal trajectories and ambitious goals. Symbolic status elements proved more influential than material rewards. Participant 2 stated: “*When I see nurses in higher positions and the respect they receive, I genuinely feel envious*… *The salary increase isn’t even important to me - it is the professional status. Even the way senior nurses look at me motivates me to elevate my own position. I keep telling myself, if they can achieve it, so can I. My envy is directed at their job title, the respect from visitors and managers - this pushes me to advance.*”

#### 4.1.4. Leadership and Personality Qualities

Leadership and personality qualities were associated with benign envy through observable traits through observable traits related to interpersonal presence and executive demeanor—specifically, emotional composure, intellectual depth, professional gravitas, charisma, and personality maturity. These qualities reflect how individuals present themselves in supervisory roles and influence others through their personal style. For instance, Participant 4 noted: *“The most remarkable quality my colleague possesses is his dignified presence and integrity–though I should clarify I’m describing this as a female observer. He is exceptionally intelligent and principled, with a visible sense of calm in his expression and a natural charisma that commands attention.*”

#### 4.1.5. Positive Moral and Behavioral Virtues

Positive moral and behavioral virtues featured in accounts of benign envy when colleagues demonstrated ethical conduct, moral integrity, and selfless dedication under pressure, patience, and prosocial behaviors—qualities rooted in moral character rather than leadership style. Participant 5 recalled: *“During the peak of COVID-19, I had a colleague who worked far beyond capacity. You could see exhaustion in the eyes, yet they never showed it. They’d even tell us to rest while covering our duties. I often looked at them wistfully, wishing I could be that strong to comfort others in such conditions*.*”* Participant 14 added: *“Some colleagues’ ethics and skills are so renowned that the entire hospital knows and respects them. I deeply admire their demeanor and consciously emulate them, feeling that positive envy.*”

#### 4.1.6. Existential‐Spiritual Awareness

Existential‐spiritual awareness was associated with benign envy through colleagues’ transcendent perspectives on mortality and purpose, especially during crises. Participant 10 recounted: “When COVID‐19 overwhelmed our hospital, a colleague volunteered for the dedicated unit. When I asked if she feared infection, she replied, “Death is truth—better it happens in the right way.” Later, learning of her critical condition, I realized she *might be closer to God, having understood true humanity. Eventually, I, too, volunteered, thinking ‘If she could transcend self-preservation, so could I.’ Witnessing her struggle on the ventilator, I envied her spiritual awareness and death acceptance.*”

#### 4.1.7. Proactive Striving for Advancement

Proactive striving for advancement was associated with benign envy through participants’ observations of colleagues’ persistent pursuit of growth despite obstacles. Participant 25 described: *“My colleague commuted from another city under difficult circumstances, yet she never ceased seeking better opportunities. When she shared that she had secured a well-paying part-time position locally, I felt genuinely inspired rather than resentful. Her perseverance despite all obstacles made me reflect on my own approach to professional challenges.”*

#### 4.1.8. Effective Therapeutic Communication Skills

Effective therapeutic communication skills were associated with benign envy through colleagues’ success in building meaningful patient connections and consequent popularity. This theme focuses specifically on interpersonal and relational abilities—such as active listening, empathy, patient education, and building trust. Participant 8 noted: *“I often ask them how they become so beloved by patients*… *It’s obvious when shift changes happen—patients specifically ask for certain nurses or openly say,* ‘*That nurse is really good!’ I try to be one of those nurses that patients genuinely like.”* Similarly, Participant 5 described a formative experience: *“What triggers my benign envy is seeing my colleagues solve patient issues in unconventional ways—ways I haven’t considered. They can connect with patients much better than I can, and I feel frustrated that I can’t interact in the same way. I always see them as more successful than me.”*

### 4.2. Perceived Drivers of Benign Envy Among Nurses

#### 4.2.1. Self‐Evaluation Coupled With Intrinsic Motivation Dynamics

Self‐evaluation coupled with intrinsic motivation dynamics was described in participants’ accounts as an influence on how they responded to benign envy. Individuals with strong self‐evaluation tendencies and internal drive experienced benign rather than malicious responses. Four patterns were evident in participants’ perceptions: belief in growth capacity, acknowledgment of skill gaps, recognition of improvement needs, and acceptance of disparities. Participant 1 stated: *“I’m perfectionistic by nature and value progress, but never at others’ expense. Most friends share this growth mindset.”* Participant 24 noted: *“Everyone’s career reflects their efforts. If someone outperforms me, it’s either my unmet potential or their deserved achievement.” Participant 21 exemplified: “I don’t resent skilled colleagues because I believe I can achieve similar competence through self-improvement. Any current gap reflects my past choices, not permanent limitations.”*

#### 4.2.2. Professional Modeling Coupled With Constructive Competition

Participants described professional modeling and constructive competition in relation to emulation, goal‐oriented rivalry, positive regard, and nonharmful advancement. Participant 4 explained: *“I believe everyone should pursue their own path while drawing inspiration from colleagues. Whether emulating a coworker’s educational pursuits or adopting another’s interpersonal skills, these serve as constructive models - never as justification for sabotaging others’ careers.”* Participant 25 employed a botanical metaphor: *“The concept of positive envy is perfectly illustrated by the example of a red rose bush. When you see one, you naturally admire it and think, ‘I wish this was in my garden.’ However, you never break or destroy it. Instead, you take a cutting to cultivate one for yourself, hoping to have such a flower someday. This is exactly what benign envy is*. *You admire something and strive to obtain a measure of it for yourself through your own efforts, rather than by destroying the role model.”* Participant 1 added: *“Due to my circumstances, I had to be strategic in my career planning. As a result, I methodically observed my successful colleagues, identified their effective habits, and emulated them to build my own path to success.”*

#### 4.2.3. Ethically–Spiritually Rooted Influences

Ethically–spiritually rooted influences were described in participants’ accounts in relation to moral principles and transcendent beliefs shaped by upbringing and religious values. Participant 14 recalled: “I was raised in *a devout family that emphasized good intentions. My father always advised, ‘Never block others’ blessings if you want God to provide yours.’ This advice consistently guides me toward positive aspects of envy.”* Participant 19 stated: *“My religious family prioritized honest labor. My father insisted, ‘One must eat from their own efforts’ - this philosophy taught me to appreciate good people and emulate rather than resent them.”* Participant 18 summarized: *“My father’s constant advice was, ‘Strive to be your best self by learning from others’ experiences, not by pulling them down to elevate yourself*”.

## 5. Discussion

In this study, we explored nurses’ experiences of benign envy, with particular attention to the perceived triggers described in their accounts and the drivers they identified in relation to their constructive responses. Our findings offer several theoretical and practical implications.

### 5.1. Theoretical Implications

The findings substantially advance the current understanding of benign envy within nursing contexts. First, this study broadens the range of situations and qualities that participants associated with benign envy in nursing practice. While prior research has predominantly framed benign envy as a product of upward social comparisons in achievement contexts [33, 34], our findings revealed that nurses experience benign envy through moral admiration (e.g., ethical virtues during crises), spiritual exemplarity (e.g., existential acceptance in coronavirus disease (COVID‐19) volunteers), and relational excellence (e.g., therapeutic communication skills). This aligns with emerging work on “virtuous envy” in prosocial occupations [35], which identifies role‐modeling of moral character—not just competence—as a catalyst for constructive envy.

Second, existential‐spiritual awareness was identified as a distinct theme associated with benign envy among nurses, particularly in high‐stakes clinical environments. These findings align with Polat et al.’s [5] demonstration that self‐efficacy enhances constructive envy. Our findings suggest that participants also interpreted spiritual qualities as relevant to their experiences of constructive envy observing colleagues who demonstrated profound spiritual grounding (e.g., *“Death is truth—better it happens in the right way,”* Participant 10). This spiritual dimension may be particularly salient in caregiving professions, where existential awareness functions alongside self‐efficacy as distinct yet complementary resources. While self‐efficacy reflects confidence in one’s abilities, spiritual awareness involves meaning‐making and worldview. In our data, these two dimensions co‐occurred: nurses who observed colleagues’ existential groundedness reported both inspiration and increased professional confidence. These accounts suggest that spiritual awareness and self‐efficacy may be experienced as complementary resources in high‐stakes clinical environments although their relationship requires further investigation.

Third, leadership and personality qualities constituted a distinct theme associated with benign envy among nursing professionals. This finding is consistent with research on relational and social comparison dynamics in workplace envy. For instance, Gan [7] demonstrated how envy’s relational nature can foster positive behaviors like admiration and emulation in professional settings. Notably, our findings expanded current theories by revealing that interpersonal characteristics—particularly emotional composure emotional composure, intellectual depth, and professional gravitas—serve as critical factors in eliciting benign envy. This complements prior studies focusing on tangible factors like self‐efficacy [5], suggesting that intangible qualities such as calm demeanor and authentic engagement equally motivate constructive emulation. Collectively, these findings broaden consideration of benign envy to include personality‐related and relational qualities within high‐stakes healthcare environments.

Fourth, the findings extend the theoretical scope of benign envy by highlighting domains that were salient in participants’ accounts beyond those emphasized in classical models. Foundational theories of benign envy, such as the influential model proposed by van de Ven et al. [15], primarily focus on upward social comparisons in domains such as superior performance, money, or career advancement. While this model helps explain envy in competitive, achievement‐oriented contexts, it conceptualizes these domains as arenas in which social comparisons occur rather than as an exhaustive set of situations associated with envy. Our findings highlighted additional domains in nursing: existential‐spiritual awareness (e.g., a colleague’s conscious acceptance of mortality) and observed positive moral virtues (e.g., selflessness and unwavering dedication) were salient in participants’ descriptions of benign envy. This distinction is important because it illustrates that benign envy may be experienced not only in relation to tangible achievements but also in response to perceived moral excellence and existential depth—domains that may be particularly salient in caregiving professions.

Importantly, our findings did not suggest that these triggers have been entirely absent from previous research. Prior studies have indeed conceptualized benign envy as a source of inspiration and motivation for professional growth [36] and have linked it to the belief in the moral virtue of hard work and dedication [37]. What this study contributes is a contextually grounded account of how nurses described existential–spiritual awareness and observed moral virtues in relation to their experiences of benign envy. In line with prior research, benign envy is positively related to task performance, job engagement, and sales performance [9, 10]. These findings align with the classical emphasis on achievement‐related domains, yet they do not exhaust the possible triggers of benign envy. By identifying spiritually and morally grounded triggers, our study complements this body of work and aligns with emerging research in positive organizational scholarship that highlights the role of “elevation”—an emotional response to moral beauty—as a motivator for prosocial behavior [38].

Fifth, the findings offer a critical refinement to Social Comparison Theory [39] as applied to high‐stakes professional environments. The classic theory posits that individuals engage in upward comparisons to evaluate themselves. However, our data refined this by highlighting that the mere act of upward comparison does not deterministically lead to benign envy; participants’ accounts highlighted perceived attainability as an important aspect of how they interpreted upward comparison. This concept, explored in later refinements of the theory, suggests that comparisons are most motivating when the target’s success seems within reach [40]. Extending this, research demonstrates that comparisons with moderately successful targets—those who are slightly ahead—can be particularly powerful in boosting motivation, precisely because their success appears attainable and the gap to be bridged feels manageable [41]. In our study, this was reflected in the notion of “Relatable Excellence,” whereby successful colleagues were described not as distant or threatening figures but as accessible models who could share knowledge and pathways. The recurring participant sentiment of “if they can achieve it, so can I” illustrates how perceived attainability and access to guidance shaped participants’ interpretations of upward comparison. These findings suggest that relatable role models may provide an important context for constructive responses to benign envy although this interpretation should be examined further in future research.

Finally, the themes identified in this study provide a basis for an interpretive account of how participants described experiences of benign envy and their subsequent responses. Rather than treating the eight themes as objectively causal triggers and the three themes as independently established psychological mechanisms, the analysis suggests an interconnected pattern in participants’ accounts. Situations involving outstanding competencies, career advancement, moral qualities, relational skills, and other valued characteristics were described as triggers of benign envy in participants’ accounts. Participants then described interpreting these triggers through self‐evaluation, perceived capacity for growth, professional modeling, constructive competition, and ethical–spiritual values. These interpretations in turn drove responses described in their narratives, such as systematic observation, identification of effective habits, and constructive emulation [42]. Figure 1 presents this interpretation as a conceptual representation of the relationships described by participants rather than as a tested causal model. The account, therefore, complements existing work by illustrating how benign envy may be understood by nurses as part of a broader process involving triggers, interpretation, drivers, and constructive professional response.

**FIGURE 1:**
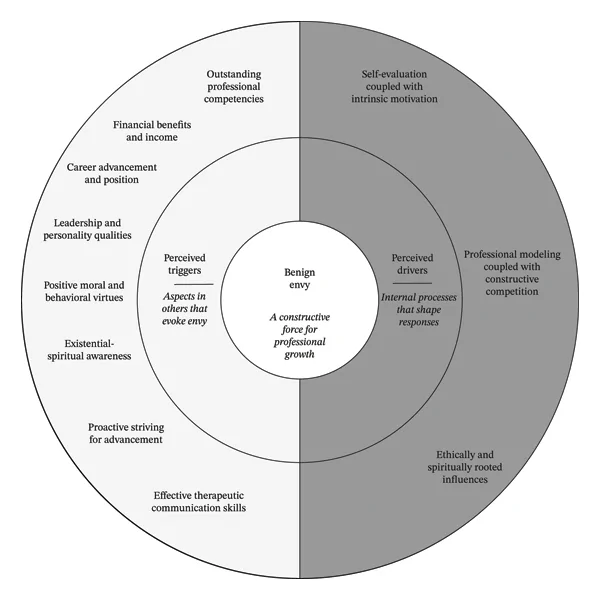
Interpretive thematic representation of perceived triggers and drivers of benign envy among nurses.

Culturally, our findings reflected the Iranian nursing context, where participants’ accounts reflected the Iranian nursing context, including references to religious and ethical values of envy as admiration rather than resentment. This cultural grounding should be considered when assessing the transferability of these interpretations to secular or more individualistic settings.

### 5.2. Practical Implications

This study provides several practice considerations for supporting constructive responses to upward comparison within nursing contexts. Because these implications are derived from participants’ accounts rather than from tested interventions, they should be regarded as areas for consideration and further evaluation rather than established strategies for producing benign envy.

First, for nursing managers and healthcare administrators, the findings suggest the value of transparent and accessible pathways for professional advancement that allow nurses to identify attainable examples of professional development. Structured mentorship programs that pair less experienced nurses with colleagues who exemplify “Relatable Excellence”—such as clinical champions or communication experts—may provide opportunities for observational learning and professional modeling consistent with participants’ accounts [40]. Furthermore, recognizing not only clinical outcomes but also demonstrable moral virtues, leadership qualities, and peer‐support behaviors may help make diverse forms of professional excellence visible and valued. This approach is consistent with contemporary principles of positive organizational behavior, which emphasize employees’ strengths and positive psychological capacities [43].

Second, in nursing education and continuing professional development, the findings suggest that nurses may benefit from opportunities to reflect on constructive and nonconstructive responses to upward social comparison. Educational programs could introduce the concept of benign envy and provide opportunities to discuss cognitive appraisal and reappraisal of upward comparisons [21]. Workshops might also encourage nurses to examine the competencies of admired colleagues—such as communication techniques, clinical decision‐making processes, or leadership behaviors—and translate relevant observations into individualized professional‐development goals. Such activities would be consistent with participants’ descriptions of self‐evaluation and professional modeling, while avoiding the assumption that benign envy can be directly produced through training.

Finally, at the individual and team level, nurse leaders may consider creating environments in which professional admiration, peer learning, and constructive comparison can be discussed openly. Leaders can model this approach by acknowledging colleagues’ strengths and sharing their own learning experiences. Regular peer‐sharing sessions, such as “clinical pearls” meetings in which nurses present challenging cases they have witnessed colleagues handle effectively, may facilitate knowledge sharing and professional learning. Such practices could make professional competencies visible within a supportive context and may help nurses interpret comparison in ways that emphasize mutual development rather than interpersonal threat. Attention to psychological safety may further support environments in which professional learning and constructive competition can occur without undermining collegial relationships [44]. These practice considerations should be evaluated empirically before conclusions are drawn about their effects on benign envy or professional outcomes.

### 5.3. Limitations and Future Directions

This study has limitations. First, the reliance on self‐reported accounts from nurses in Iran limits the transferability of the findings to other cultural and healthcare contexts. Cultural norms specific to the Iranian healthcare system may influence how nurses describe and interpret benign envy, suggesting the need for cross‐cultural studies in different settings. Second, although qualitative approaches provide insight into nuanced experiences and patterns of meaning, the present study does not establish the prevalence of particular situations associated with benign envy or statistical relationships between these experiences and professional development or well‐being. Future quantitative research could examine the prevalence of the identified themes and test potential relationships among benign envy, its associated experiences, and relevant professional outcomes.

Promising avenues for future research emerge from these findings. One direction is to investigate the consequences that participants associated with benign envy at individual and organizational levels. For example, future research could examine whether emulation associated with benign envy is related to career advancement, enhanced performance, or job satisfaction. Further research in other high‐stakes, team‐based professions, such as medicine, could examine the extent to which the themes identified in this study are transferable across occupational contexts. Longitudinal research could also explore how experiences and interpretations of benign envy change over time and how they may relate to resilience, professional development, and well‐being, particularly during periods of crisis.

Additionally, the gender distribution of our sample may limit the transferability of findings, as male nurses were overrepresented. While this composition reflects the actual demographic makeup of nursing staff in the selected hospitals—particularly in intensive and emergency units—future research with more gender‐balanced samples would help explore potential gender‐related variations in experiences and interpretations of benign envy.

## 6. Conclusion

This qualitative study explored nurses’ experiences of benign envy, with particular attention to the situations and qualities they associated with these experiences and the influences they described in relation to constructive responses. Participants described benign envy in relation to outstanding professional competencies, moral and behavioral virtues, career advancement, financial benefits, leadership and personality qualities, existential–spiritual awareness, proactive striving, and therapeutic communication skills. They also described self‐evaluation and intrinsic motivation, professional modeling and constructive competition, and ethical–spiritual influences as important drivers of how they interpreted and responded to upward comparison.

Overall, the findings portray benign envy as a multifaceted experience embedded in nurses’ professional, relational, moral, and existential contexts. Participants’ interviews suggested that upward comparison could be experienced constructively when accompanied by self‐reflection, perceived attainability, professional learning, and value‐based responses. The findings, therefore, provide a contextually grounded thematic account of how nurses make sense of benign envy and how they describe the role of triggers and drivers in relation to constructive responses. These insights may inform nursing‐management discussions concerning mentorship, professional role modeling, recognition of diverse forms of excellence, and environments that support constructive professional comparison.

## Author Contributions

Mehdi Golverdi: conceptualization, methodology, data curation, writing–original draft, and writing–review and editing. Mohammad Sadegh Sharifirad: conceptualization, supervision, formal analysis, investigation, and writing–review and editing.

## Funding

No funding was received.

## Disclosure

The authors have nothing to report.

## Conflicts of Interest

The authors declare no conflicts of interest.

## Data Availability

The data supporting the findings of this study are available from the corresponding author upon reasonable request. The data are not publicly available due to privacy and ethical restrictions.
